# Dragon’s Blood Sap: Storage Stability and Antioxidant Activity

**DOI:** 10.3390/molecules23102641

**Published:** 2018-10-15

**Authors:** Juan D. Escobar, Cristina Prieto, Maria Pardo-Figuerez, José M. Lagaron

**Affiliations:** 1 R&D Department, Bioinicia S.L. Calle Algepser 65, 46980 Paterna, Spain; jdescobar@bioinicia.com (J.D.E.); mpardo@bioinicia.com (M.P.-F.); 2 Novel Materials and Nanotechnology Group, Institute of Agrochemistry and Food Technology (IATA), Spanish Council for Scientific Research (CSIC), Calle Catedrático Agustín Escardino Benlloch 7, 46980 Paterna, Spain

**Keywords:** Dragon’s Blood Sap, proanthocyanidins, storage stability, antioxidant activity, FT-IR, UV-Vis spectrophotometry

## Abstract

Currently, consumers are demanding additive-free, fresher, and more-natural products. Dragon’s Blood Sap (DBS), the deep red latex of the specie of tree Croton lechleri (Müll. Arg.), contains a high concentration of phenolic compounds of great interest for the food, pharmaceutical, and cosmetic industries. These chemical compounds are highly susceptible to degradation. Therefore, DBS storage stability and its photo-oxidation was studied by Fourier transform infrared spectroscopy (FT-IR) and UV-Vis spectrophotometry for 39 days at different temperatures (4–21 °C) and relative humidities (0–56%), as well as under UV light exposure. It was observed that the degradation of phenolic compounds was reduced at 0% relative humidity (RH), not showing a significant effect of temperature in the range studied. UV light irradiation degraded DBS in a 20%. DBS has an exceptional high and stable antioxidant content (≥93% inhibition percentage of DPPH), which makes it a unique property to consider the DBS as an antioxidant agent or ingredient for consumer products formulations.

## 1. Introduction

Phenolic compounds are one of the most widely occurring groups of phytochemicals. This group of bioactive compounds containing one or more aromatic rings with one or more hydroxyl groups attached to them is divided into the following classes: Phenolic acids (hydroxybenzoic acids and hydroxycinnamic acids), flavonoids (flavonols, flavones, flavanols, flavanones, isoflavones, and proanthocyanidins), stilbenes, and lignans [1,2]. These compounds are secondary plant metabolites involved in the defense against ultraviolet radiation or aggression by pathogens, contributors to plant pigmentations and antioxidants, as well as responsible for their organoleptic properties [1,3]. Additionally, these compounds have also demonstrated a huge number of potential benefits for human health, related to their presumable role in the prevention of various diseases associated with oxidative stress, such as cancer, cardiovascular, and neurodegenerative diseases [4,5]. Fruits, vegetables, leguminous plants, and cereals are good natural sources for polyphenols. The content of polyphenols varies significantly as a function of several parameters including genetic factors, environmental factors (climate, agronomic factors), manner of cultivation, and ripeness [1]. In recent years, special attention has been paid to the isolation of phenolic compounds from different raw materials, especially from inexpensive or residual sources (medicinal plants, fruits, vegetables, industrial by products, and beverages).

Dragon’s Blood Sap (DBS) is the common name of the deep red latex that oozes out upon cutting or slashing the bark of the specie of tree Croton lechleri (Müll. Arg.), also known as Croton draco var. cordatus (Müll. Arg.), and Oxydectes lechlen (Müll. Arg.) Kuntze [6]. It is a native biodiversity species of tree from South America (Brazil, Bolivia, Colombia, Ecuador, and Peru) [7].

DBS, very well-known in traditional medicine, has been related to several therapeutic uses such as analgesic, anti-inflammatory, antibacterial, antifungal, antihemorrhagic, antimicrobial, antioxidant, antiseptic, antitumor and cytotoxic, antiulcer and antidiarrheal, antiviral, astringent, cicatrizing, immunomodulatory, muscular tissue regenerator, mutagenic and antimutagenic, purifier, skin conditioning, wound healing, among others [8,9,10]. DBS bioactivity is due to the high content of proanthocyanidins (>90% dry weight of the resin), but also to its content in taspine, catechin, epigallocatechin, epicatechin, and a small percentage of terpene compounds [11,12].

The interest in DBS relies on the high content of polyphenols, especially proanthocyanidins, one of its main constituents, which, due to their low water solubility or poor bioavailability, constitute the only dietary group of antioxidants present in the colon, which is particularly exposed to oxidizing agents and may be affected by inflammation and numerous diseases such as cancer [5,13,14,15]. However, DBS could be involved in other industrial applications. Currently, consumers are demanding additive-free, fresher, and more-natural products. In this sense, polyphenols may be used as natural antioxidants, natural colorants, or preservatives for foods. Moreover, they could be used as additives in the production of paints, paper, cosmetics, and pharmaceutical products, replacing current synthetic additives, some of which are being restricted due to their carcinogenicity [4]. Taking into account the beneficial health effect offered by these compounds, their incorporation in food, cosmetics, pharmaceutical, or agricultural products will represent an added value.

Hitherto, special attention has been paid to the therapeutic potential of DBS [8,9,10,15,16], its antimicrobial activity [17,18], and its chemical composition [9,12,19,20,21,22]. However, little work was done on the study of its stability. It is well known that these bioactive compounds are highly susceptible to degradation. During the different processing stages, storage, and consumption, polyphenols could be subjected to different stress conditions such as high temperatures, light, oxygen, solvents, presence of enzymes, proteins, metallic ions, or associations with other food constituents, which can damage their structures and thus reduce their beneficial activity [4].

Therefore, the aim of this work is to study the storage stability at different conditions of temperature and humidity and the photo-oxidation of DBS, as well as the effect of these stress conditions on its antioxidant activity.

## 2. Results and Discussion

DBS stability was studied considering several storage conditions, under different temperature and relative humidity (% RH) environments. Additionally, an accelerated ageing treatment was carried out subjecting the freeze-dried DBS to UV light irradiation. The damage of DBS was measured by UV-Vis spectrophotometry and Fourier transform infrared spectroscopy technique (FT-IR). Moreover, the effect of these stress conditions on the DBS antioxidant activity was evaluated.

### 2.1. DBS Stability by UV-Vis

**Figure 1:**
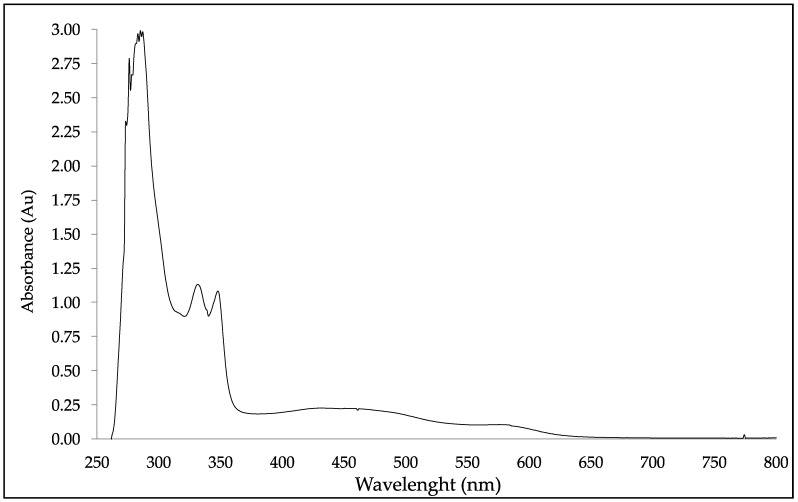
UV-Vis spectrophotometry screening spectra of Dragon’s Blood Sap (DBS) at a concentration of 0.5 mg/mL.

UV-Vis spectra of DBS samples were collected and analyzed by UV-Vis spectrophotometry. DBS presented four UV-Vis bands at a maximum absorption wavelength (λ_max_) of 331 nm, 347 nm, 464 nm, and 579 nm, as it is shown in Figure 1. The complex nature of the DBS’ spectrum makes the detailed theoretical analysis difficult [13]. Nevertheless, some of the later λ_max_ could be related to important bioactive materials, such as the quinoxaline, which has a UV absorption at λ_max_ of 242 and 331 nm; and 6-thioguanine has UV absorption at a λ_max_ of 258 and 347 nm [23]. The quinoxaline is a fused heterocycle of benzene and pyrazine rings with a wide array of pharmacological activities viz. anti-cancer, antimalarial, anti-inflammatory, antimicrobial, antiHIV, etc. [23,24]. The 6-thioguanine is an antimetabolite of purine analogue type, used in the treatment of acute leukemias due to its inhibition of DNA synthesis in cancer cells [25,26].

#### 2.1.1. DBS’ Stability According to Relative Humidity (% RH)

**Figure 2:**
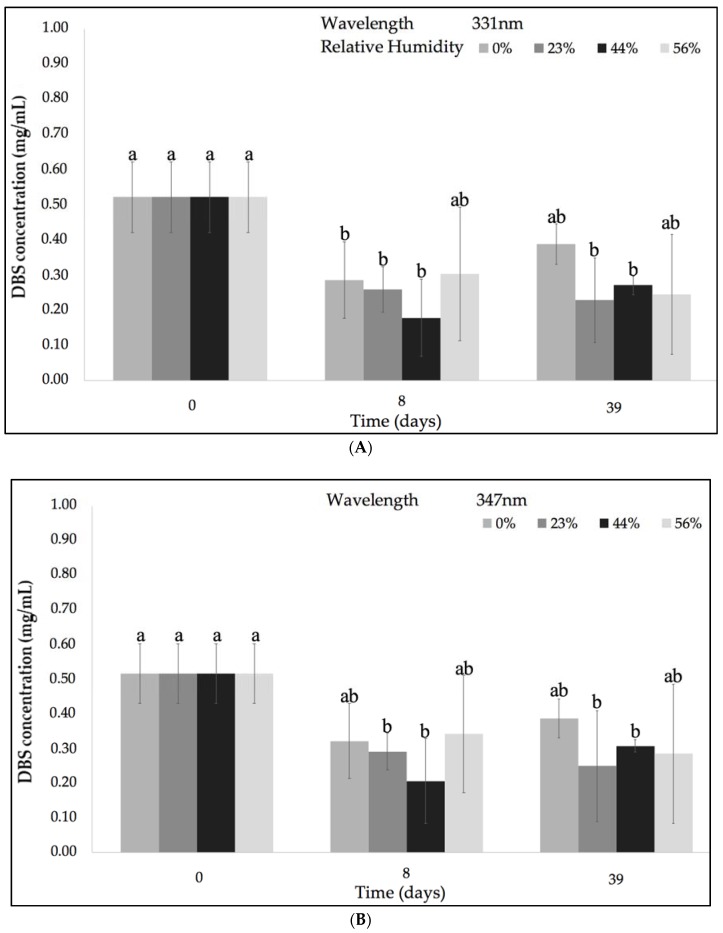

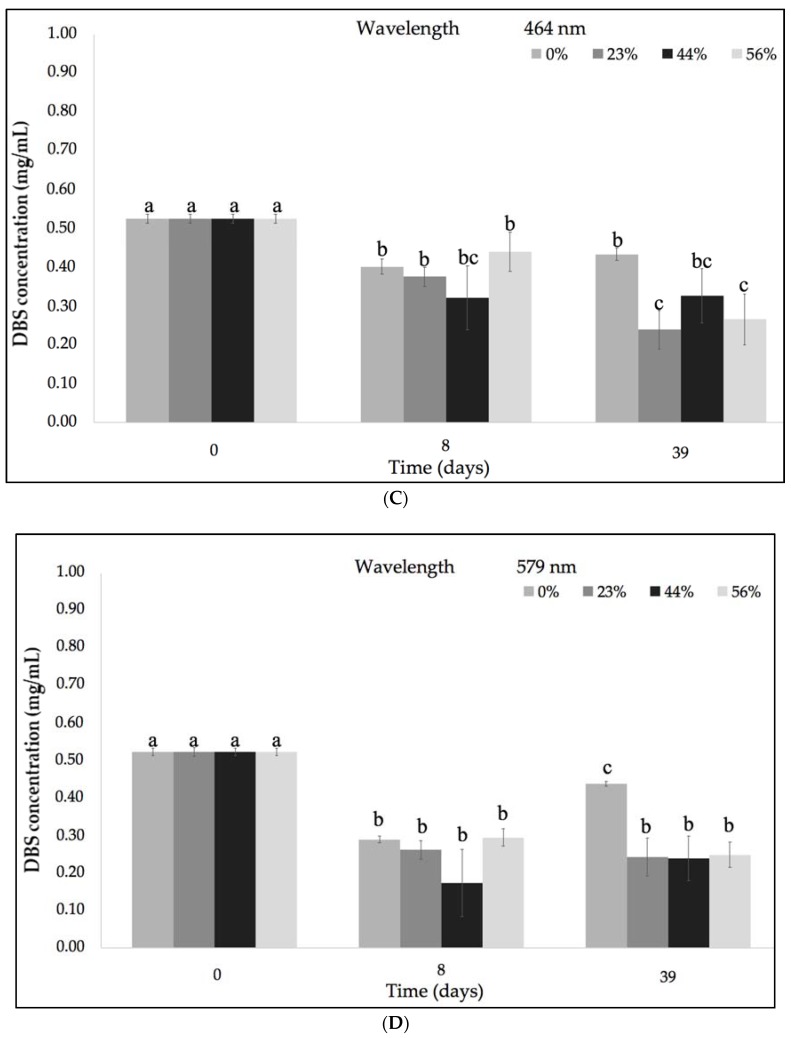
Evolution of the DBS concentration under various % relative humidity (RH) and ambient temperature (~21 °C) after 39 days of storage according to the different absorption bands at 331 nm (**A**), at 347 nm; (**B**) at 464 nm; (**C**) and at 579 nm (**D**). Different letters within the graph (a–b) indicate significant differences among the conditions (*p* < 0.05). Different letters within the graph (ab, bc) indicate no differences among the samples (*p* > 0.05).

**Figure 3:**
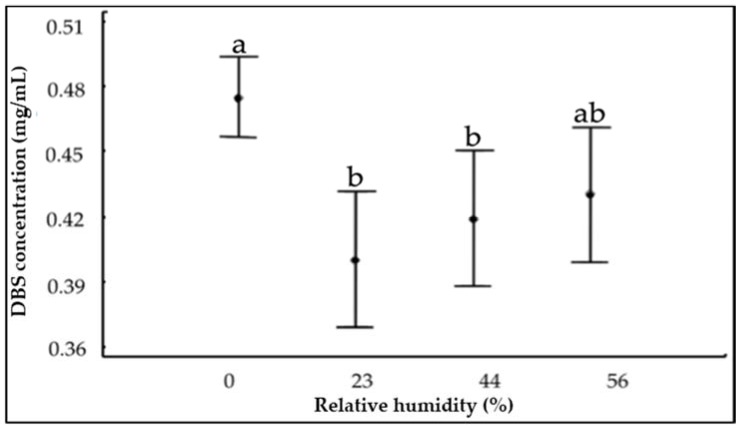
Tukey’s honestly significant difference (HSD) test of the evolution of Dragon’s Blood Sap concentration after 39 days of storage at 21 °C and different relative humidity. Different letters within the graph (a–b) indicate significant differences among the conditions (*p* < 0.05). The ab value indicates no differences among the conditions (*p* > 0.05).

Figure 2 represents the variation of the concentration of the DBS compounds at various % RH and ambient temperature (~21 °C) after 39 days of storage at the UV-Vis λ_max_ identified in Figure 1. All the experimental conditions of % RH produced significant differences between the initial (0.52 ± 0.02 mg/mL) and final concentration of DBS samples. In order to clarify the effect of the % RH on DBS stability, a Tukey’s honestly significant difference test (HSD) was performed and it is shown in Figure 3. Samples stored under 0% RH were more stable when compared with the other three % RH conditions (23%, 44%, and 56%). Lavelli et al. found that the degradation kinetics of grape skin phenolics could be reduced by decreasing the water activity in the grape skin powder [27]. They attributed this increased stability to a limited water mobility and diffusion rate of reagents, as well as to a decreased enzyme activity [27].

#### 2.1.2. DBS’ Stability According to Temperature

**Figure 4:**
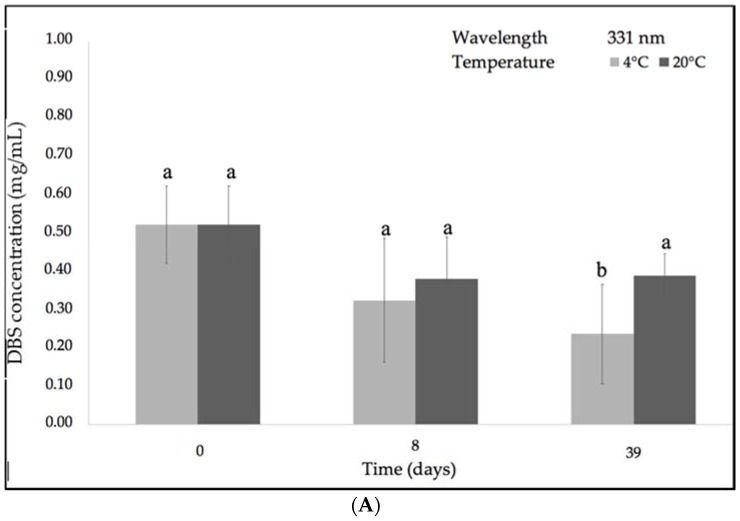

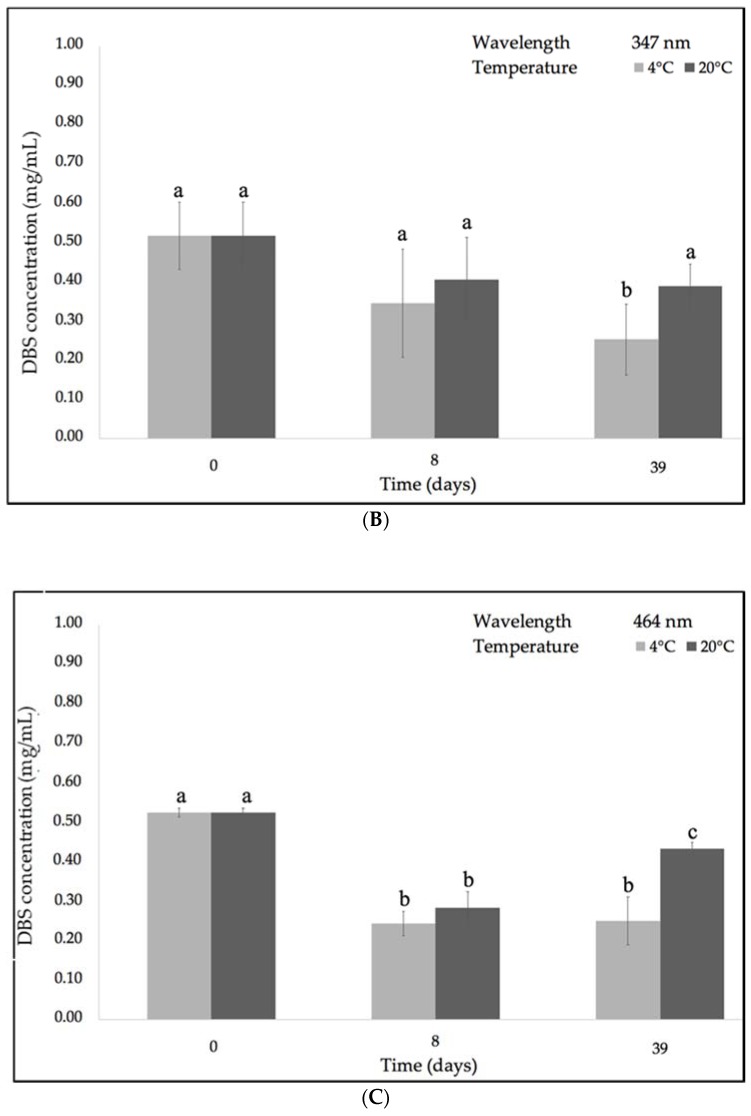

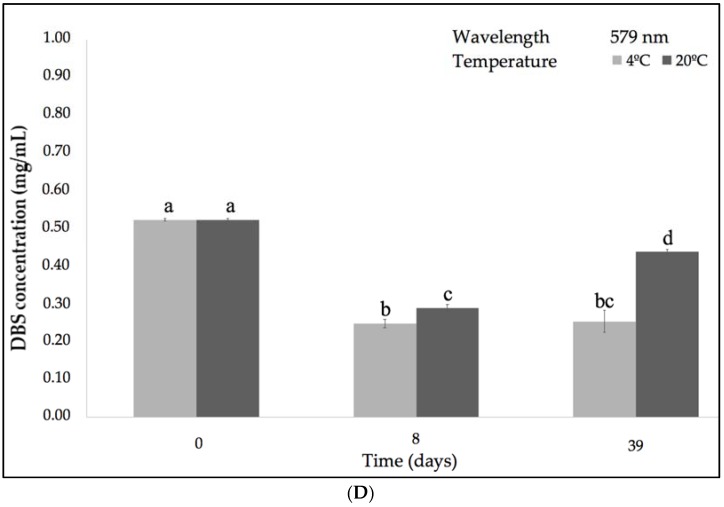
Evolution of DBS concentration after 39 days of storage under low (4 °C) and ambient temperature (~21 °C) and 0% RH, according to the different absorption bands: (**A**) 331 nm; (**B**) 347 nm; (**C**) 464 nm; (**D**) 579 nm. Different letters indicate significant differences among the samples (*p* < 0.05).

DBS stability considering the storage temperature (4 °C and ~21 °C) at the different wavelengths is represented in Figure 4. Similar to the previous analyses of DBS’ stability according to relative humidity (% RH), certain differences between the initial (0.52 ± 0.02 mg/mL) and final concentrations of all DBS samples were also identified in reference to both experimental temperatures. DBS components that absorbed at the lower wavelength region were not significantly changed. However, in the case of the DBS components that absorbed within the higher region, a reduction in DBS concentration was observed at 4 °C when compared with ambient temperature. In addition, it was observed that after one week, the DBS concentration was reduced, but at the end of the experiment, the DBS concentration increased. This phenomenon could be due to the complex nature of DBS, which could interact at ambient temperature and produce other secondary metabolites absorbing in the long wavelength region.

**Figure 5:**
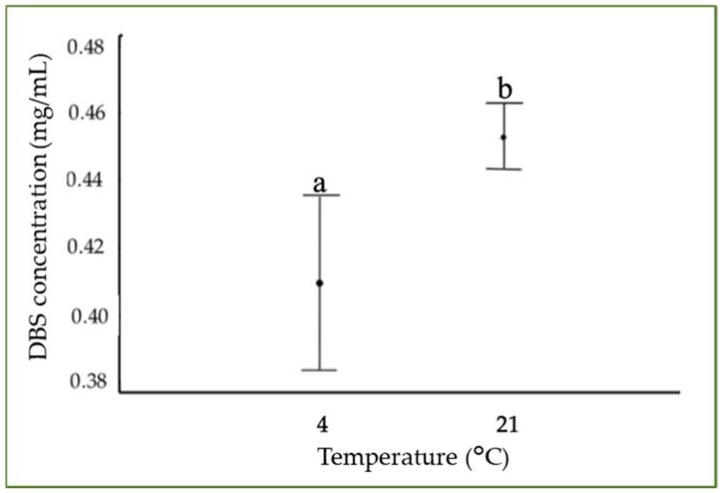
Tukey’s test of the evolution of Dragon’s Blood Sap concentration at different storage temperatures and 0% RH. Different letters indicate significant differences among the samples (*p* < 0.05).

Figure 5 represents the Tukey’s HSD test (honestly significant difference) in order to clarify the effect of the temperature on the concentration of DBS samples. With a level of 95.0% confidence, a statistically significant difference was observed between 4 °C and 21 °C, presenting a higher stability at ambient temperature. Kopjar et al. observed a higher stability of the polyphenols present in the sour cherry puree extracts at 4 °C than at room temperature [2]. Xu et al. found slower degradation kinetics for the proanthocyanidins dimers at temperatures lower than 25 °C than at 40 °C [28]. Nobrega et al. studied the effect of the hot air during a drying process finding that temperatures higher than 60 °C contributed to the degradation of phenolic compounds and consequently to a reduction in the antioxidant activity [29].

#### 2.1.3. DBS’ Stability at Ultraviolet (UV) Light Exposition

**Figure 6:**
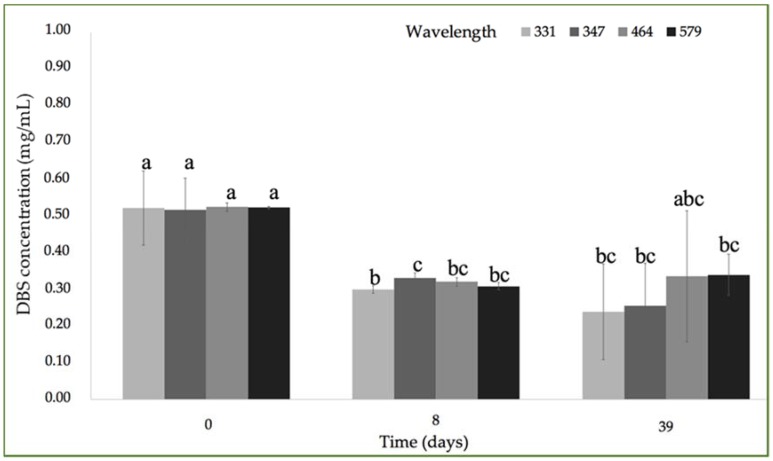
Evolution of DBS concentration after 39 days of storage under UV-light according to the different absorption bands: 331 nm; 347 nm; 464 nm; 579 nm. Different letters indicate significant differences among the samples (*p* < 0.05). Different letters within the graph (abc, bc) indicate no differences among the samples (*p* > 0.05).

Figure 6 shows the effect of UV light on the DBS concentration. The radiation produced by UV light exposure produced a reduction into the DBS concentration, from an initial 0.52 ± 0.02 mg/mL to a final concentration of 0.32 ± 0.21 mg/mL for DBS compounds absorbed at lower UV-Vis λ_max_ region, and to a concentration of 0.48 ± 0.09 mg/mL in the case of DBS compounds absorbed at the higher UV-Vis λ_max_ region. Masek et al. observed that UV irradiation affected quercetin stability in a higher extent than temperature [30]. Other authors have found that the effect of UV light caused a higher degradation than temperature in a copigment of anthocyanin and polyphenols complex [31].

The complex nature of the DBS makes the proper description of the chemical behavior of DBS under the different storage conditions difficult. Therefore, previous results were compared with those obtained by FT-IR.

### 2.2. DBS Stability by FT-IR

**Figure 7:**
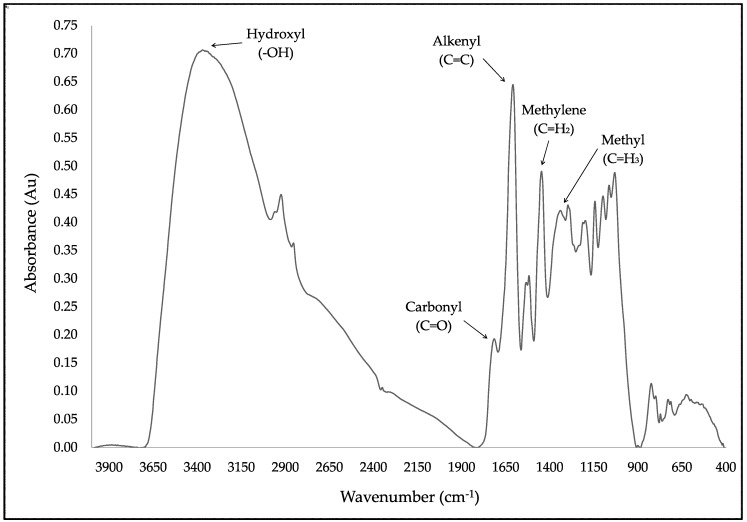
Typical Fourier transform infrared spectroscopy (FT-IR) spectrum of freeze-dried DBS. Arrows indicates the Functional Groups bands discussed in the text.

FT-IR spectroscopy was used to describe the changes of DBS composition exposed to certain storage conditions. Figure 7 represents the typical FT-IR spectrum of freeze-dried DBS samples. The spectrum of freeze-dried DBS was compared to the spectrum of natural DBS, which did not show significant differences in the chemical composition (results not shown). The DBS spectrum presented the characteristic bands of the proanthocyanidins, which are of its main constituents [20,32,33] (Figure 7). Among the bands found in the spectrum, the intense O–H stretching band between the 3700–3000 cm^−1^ could be ascribed to the formation of the hydrogen bond between phenolic hydroxyl of proanthocyanidins. The absorption bands between 1100 and 1600 cm^−1^ were attributed to the polyflavonoids moiety, and the absorption bands between 700–1500 cm^−1^ were attributed to the presence of procyanidin structure [34].

The DBS spectrum (Figure 7) was characterized by the 3300 cm^−1^ band related to the hydroxyl groups (-OH), a high intense band appearing at 2920 cm^−1^, and other one at 2854 cm^−1^, corresponding to the alkyl C–H stretch (2950–2850 cm^−1^) [33]. Another band was also assigned to the carbonyl group (~1700 cm^−1^) and to the C–O bond with the absorption band around 1200 cm^−1^ [33]. The region around 1700–900 cm^−1^ was mostly related with phenolic compounds [35]. Since the absorption bands in the frame of 900 and 1100 cm^−1^ could be mainly assigned to carbohydrates [35], the study was focused on the frame of 1700–1100 cm^−1^. Within this region, there is a band at 1519 cm^−1^, which is present in large amounts in aromatic C=C bending compounds with phenyl bonds similar to those in polyphenolic compounds, such as flavonoids [36,37,38]; as well as the band observed at 777 cm^−1^, attributed to the skeletal stretching modes of the aromatic ring and CH out-of-plane deformation of the aromatic rings with two adjacent free hydrogen atoms, respectively, indicating the prominent presence of procyanidin structure [39,40].

**Figure 8:**
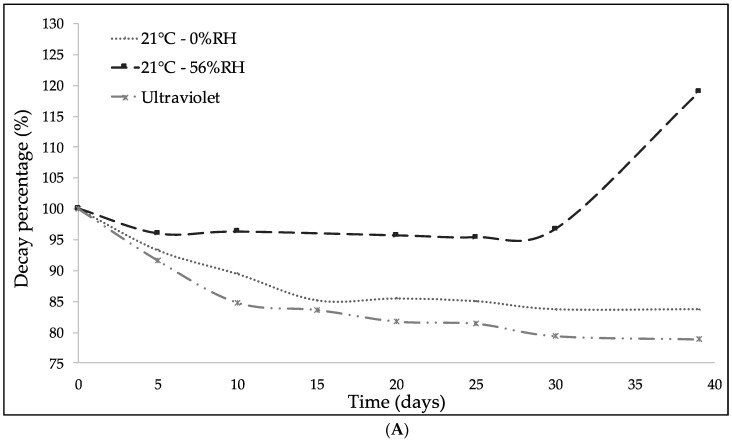

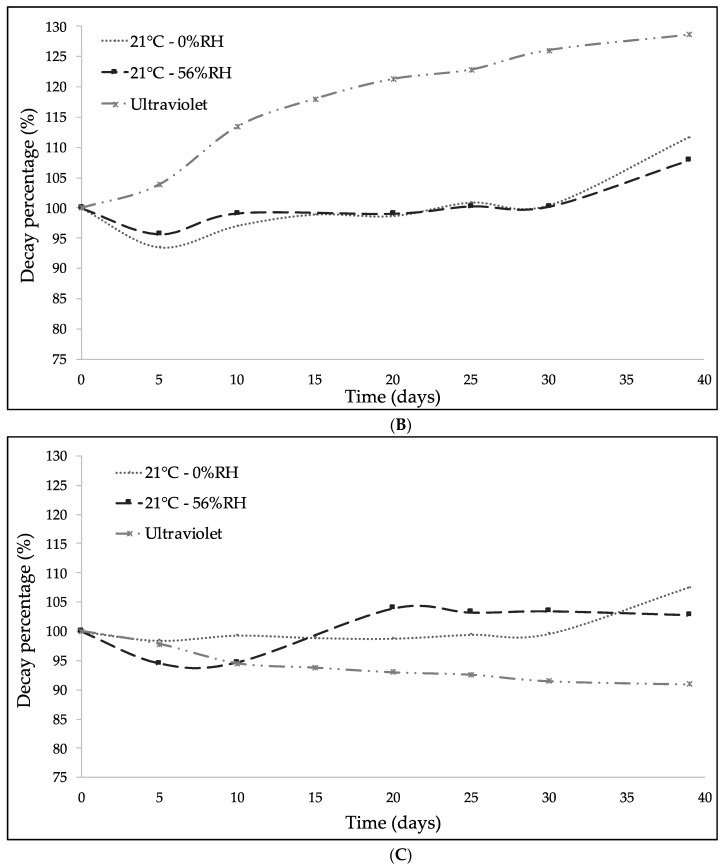

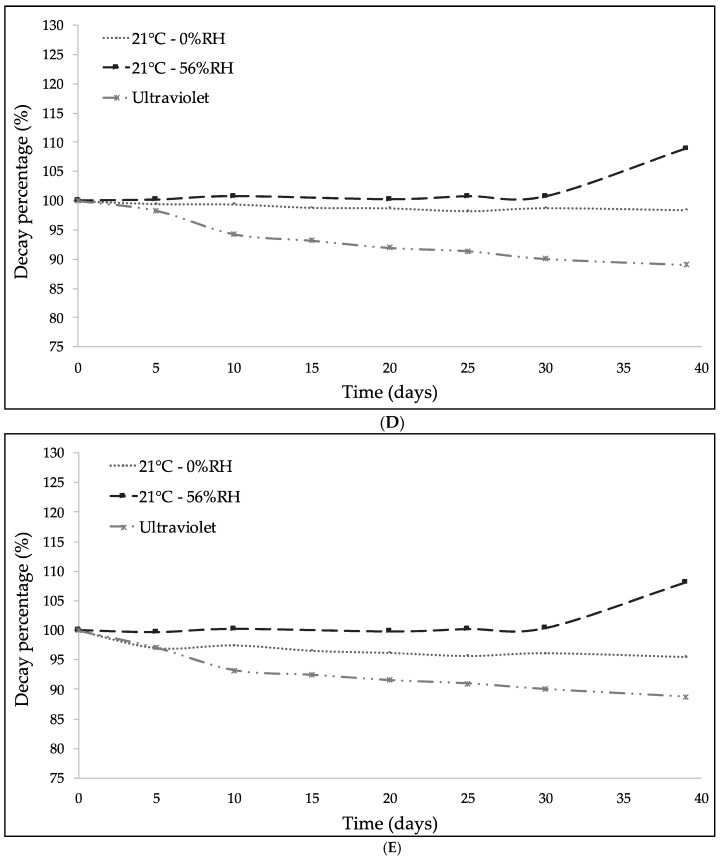
Evolution of the decay percentage of DBS characteristic functional groups with respect to the internal standard band at 1519 cm^−1^ after 39 days under different temperatures, % RH, and UV-light irradiation: (**A**) Hydroxyl group (-OH) (3300 cm^−1^); (**B**) carbonyl group (C=O) (1715 cm^−1^); (**C**) alkenyl group (C=C) (1610 cm^−1^); (**D**) methylene group (C–H_2_) (1440 cm^−1^); and (**E**) methyl group (C–H_3_) (1342 cm^−1^).

In Figure 8, the evolution of the bands with the time exposed under UV light irradiation is presented. The aromatic band at 1519 cm^−1^ was used as a reference to study the DBS stability with respect to the bands identified previously, since it remained unchanged under all conditions. Figure 8A present the hydroxyl groups (-OH) behavior. In a similar fashion to the results obtained by UV-Vis spectrophotometry, DBS presented an interaction based on the humidity of the storage media. DBS concentration increased as the % RH was increased, which could be due to the absorption of water. At 0% RH, a slight reduction of the hydroxyl groups by effect of the relative humidity was noted. The UV light radiation produced a higher reduction, compared to the other storage conditions. This reduction could be due to the effect of the UV light but also to the light bulb temperature. 

Figure 8B illustrates the effect of the storage conditions on the carbonyl group (1715 cm^−1^). An increment in the DBS absorption signal under UV light irradiation was observed. This phenomenon could be due to the biochemistry and oxidation of DBS, by which the hydroxyl groups were transformed into carbonyl groups [30].

Finally, the evolution of the alkenyl, methylene, and methyl groups is presented in Figure 8C–E, respectively. At the storage conditions considered, no significant changes were observed into the chemical structure of the sample at these absorption bands. Nevertheless, UV light irradiation produced the biggest reduction in all the studied functional groups. According to the previous results, the best conditions for DBS storage should avoid high humidity and UV light irradiation.

### 2.3. Antioxidant Capacity Stability

The structural chemistry of proanthocyanidins suggests different antioxidant properties, such as (i) high reactivity as a hydrogen or electron donor, (ii) the ability to stabilize or delocalize an unpaired electron, and (iii) the ability to chelate transition metal ions [41]. In order to define the impact of different storage conditions on the DBS antioxidant activity, spectrophotometric DPPH assay was used to study the DPPH inhibition percentage (IP DPPH (%)) of DBS.

Table 1 presents the results of IP DPPH% of the DBS samples at the studied storage conditions. All samples had a high rate of inhibition of the DPPH free radical; with values higher than 90% and stable under the studied storage conditions. These results demonstrated that DBS had an exceptional high and stable antioxidant activity, which could be really interesting for multiple applications [42].

The results obtained by UV-Vis spectrophotometry and FT-IR showed that the components of DBS were degraded under the studied storage conditions. However, its antioxidant activity was not affected. This phenomenon demonstrated the synergic effect of the complex chemical profile of DBS, which could be used for multiple industrial applications.

## 3. Materials and Methods

Dragon’s Blood Sap (DBS) was kindly provided by Q’omer BioActive Ingredients (Valencia, Spain). Natural DBS (total solids 25.95% ± 0.83%) was used as received without further purification. Samples of DBS were freeze-dried and sealed until used for storage stability and photo-oxidation tests. Saturated salt solutions of magnesium nitrate (56% RH), potassium carbonate (44% RH), potassium acetate (23% RH), silica gel, 6-hydroxy-2,5,7,8-tetramethylchromane-2-carboxylic acid (Trolox) (≥97.0%), and potassium bromide FT-IR grade (KBr) were purchased from Sigma-Aldrich (Tres Cantos, Spain).

### 3.1. Effect of the Storage Conditions and the Photo-Oxidation on DBS Stability

The DBS storage stability was studied under different conditions of relative humidity (0%, 23%, 44%, and 56% RH at 21 °C), different storage temperatures (4 and 21 °C at 0% RH), and under UV-light irradiation. This study was performed by ultraviolet-visible (UV-Vis) spectrophotometry and Fourier-Transform Infrared spectroscopy (FT-IR) comparing the spectra of freeze-dried DBS with the spectra of the samples at different conditions, which were collected every three days during a time span of 39 days.

For such study, samples of freeze-dried DBS were equilibrated in desiccators stored at different percentages of relative humidity, by using silica gel (0% RH) and oversaturated salt solutions of potassium acetate (23% RH), potassium carbonate (44% RH), and magnesium nitrate (56% RH), respectively; at cooling temperature (4 °C) or ambient temperature (~21 °C), accordingly.

An Ultra-Vitalux lamp from OSRAM Lighting S.L. (Madrid, Spain) was used to irradiate samples with ultraviolet (UV) light. This lamp operates with a power of 300 W that produces a blend of radiation very similar to that of natural sunlight, which is generated by a quartz discharge tube and a tungsten filament. The radiation of 315−400 nm after 1 h of exposure is of 13.6 W, and the radiation of 280−315 nm after 1 h of exposure is of 3.0 W [43].

Ten grams of freeze-dried DBS were placed on Petri dishes at the correspondent condition and aliquots were taken each three days for analysis. For the FT-IR analysis, KBr pellets containing the freeze-dried DBS were subjected to the studied storage conditions.

### 3.2. Ultraviolet-Visible (UV-Vis) Spectrophotometry

The UV-Vis spectral measurements were performed in a UV4000 spectrophotometer (Dinko Instruments, Barcelona, Spain). Aliquots of DBS subjected to the different study conditions were solubilized in methanol at a concentration of 0.5 mg/mL. Methanol was used as blank. Standard curves of DBS concentration were built using methanol solutions of known DBS concentration (0–0.8 mg/mL) at the different absorption bands.

### 3.3. Fourier-Transform Infrared Spectroscopy FT-IR

FT-IR spectra of DBS samples were collected using a FT-IR Tensor 37 equipment (Bruker, Rheinstetten, Germany) in the absorption mode using KBr pellets. All spectra were collected by averaging 10 scans in the range of 4.000 to 400 cm^−1^ with a 4 cm^−1^ resolution.

### 3.4. Antioxidant Capacity by DPPH Method

Spectrophotometric DPPH assay was used to study the inhibition percentage (IP) of DPPH by DBS. An aliquot of 0.1 mL of DBS methanol solutions (0.5 mg/mL) were added to 2.9 mL of DPPH (40 mg/L of DPPH in methanol), shaken in the vortex and kept in the dark for 30 min at room temperature. Sample absorbance was measured at 517 nm with UV-Vis spectrophotometer (UV4000, Dinko Instruments) using methanol as blank. The radical scavenging activity of each sample was calculated according to the Equation (1):

(1) $$\mathrm{IP}\;\mathrm{DPPH}\;(\%)=\frac{A\;\mathrm{control}-A\;\mathrm{sample}}{A\;\mathrm{control}}\times 100$$

where IP DPPH (%) is the inhibition percentage of DPPH, A control is the absorbance of pure DPPH solution, A sample is the absorbance of the sample after reacting with DPPH. Antioxidant activity was also expressed as Trolox equivalent antioxidant activity (TEAC). Standard curve at different Trolox concentrations was build. Measurements were done in triplicate.

### 3.5. Statistical Analysis

Statgraphics Centurion XV (Statistical Graphics Corp., Tulsa, OK, USA) was used for data analysis. Data were expressed as mean ± standard deviation. Firstly, analysis of variance (ANOVA) was carried out to investigate the effects of the studied parameters on the sample. Secondly, mean values were compared by using the Tukey’s honestly significant difference (HSD). Differences between means were considered at *p*-value < 0.05.

## 4. Conclusions

The DBS stability was studied by UV-Vis spectrophotometry and FT-IR under several storage conditions, taking into consideration the effect on the antioxidant activity. Results demonstrated that changes were produced on the concentration of the DBS constituents at different storage conditions. For example, the presence of moisture contributed to the DBS degradation. In case of temperature, no significant effect was detected in the range studied. However, when UV light irradiation was applied, a significant reduction of the DBS concentration took place. Nevertheless, even when changes into its chemical structure were detected, the DBS antioxidant activity remained stable under the studied storage conditions.

The low degree of degradation of samples, just a 20% when DBS was subjected to UV-light during 39 days, could be due to the protective effect exhorted by proanthocyanidins, which are one of the main constituents of DBS. In addition, the synergic effect between constituents could contribute to that stability. Therefore, the high stability observed for DBS together with its natural origin, could confer interesting characteristics in multiple industrial added-value products, such as food, pharmaceuticals, nutraceuticals or cosmetics, paints or paper products, as an antioxidant agent or as an ingredient, opening an interesting opportunity to explore new applications for DBS.

## Figures and Tables

**Table 1:** Antioxidant capacity of Dragon’s blood sap (DBS) under different environmental conditions over a period time of 39 days. Data represent the mean ± SD of triplicate assay for each sample. The mean ± SD at each storage condition followed by the same letter are not significantly different at *p* < 0.05.

Sample	Time (Days)	IP DPPH (%)
Natural DBS	0	92.76 ± 0.29 ^a^
4 °C 0 %RH	39	93.45 ± 0.35 ^b^
21 °C 0% RH	39	93.38 ± 0.28 ^b^
21 °C 23%RH	39	92.97 ± 0.50 ^b^
21 °C 44% RH	39	93.08 ± 0.19 ^b^
21 °C 56% RH	39	93.19 ± 0.19 ^b^
UV	39	93.12 ± 0.13 ^b^
